# The Role of Fullerenes in Neurodegenerative Disorders

**DOI:** 10.3390/jnt5010001

**Published:** 2024-01-16

**Authors:** Daisy L. Wilson, Jyoti Ahlawat, Mahesh Narayan

**Affiliations:** 1 Department of the Environmental Science & Engineering, The University of Texas at El Paso, El Paso, TX 79968, USA; 2 Department of Chemistry and Biochemistry, The University of Texas at El Paso, El Paso, TX 79968, USA

**Keywords:** fullerenes, carbon nanomaterials (CNMs), oxidative stress, amyloid fibrillation, neurodegeneration

## Abstract

The use of carbon nanomaterials including fullerenes, carbon nanotubes, carbon nano-onions, carbon dots and carbon quantum dots for environmental applications has increased substantially. These nanoparticles are now used in the development of sensors and switches, in agriculture as smart fertilizers and in the biomedical realm for cancer therapy intervention, as antioxidants, in gene delivery and as theranostics. Here, we review the role of fullerenes as neuroprotectants. Their sp^2^ hybridized architectures and ability to intervene in the soluble-to-toxic transformation of amyloidogenic trajectories is highlighted here, along with other physico–chemical properties that impact interventional efficacy. Also highlighted are drawbacks that need to be overcome and future prospects.

## 1. Introduction to Fullerenes

**Figure 1.**
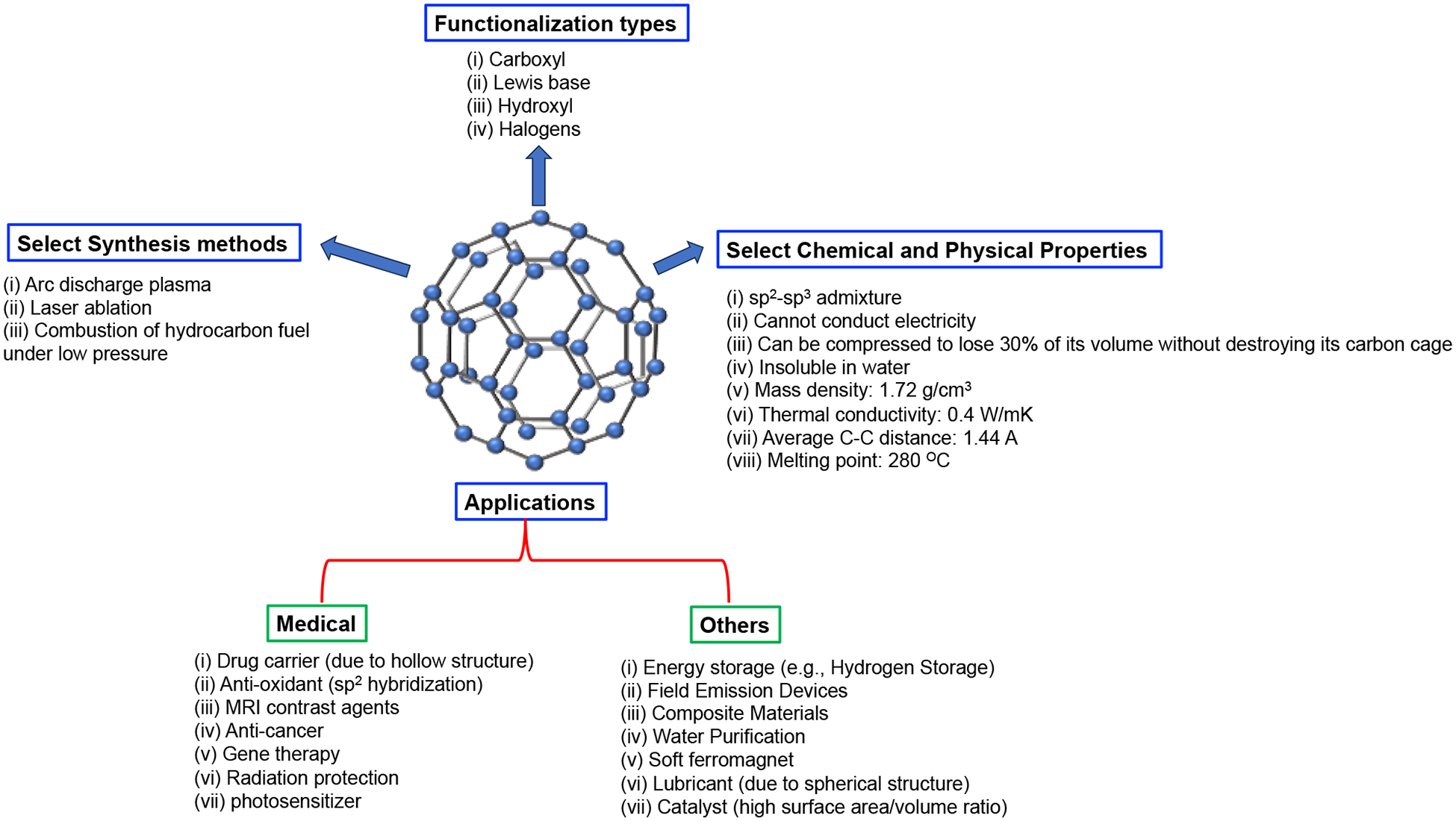
Schematic representation of C_60_ and its properties and applications.

As the first symmetric nanostructures in the family of carbon nanomaterials, the fullerenes are zero-dimensional nanoparticles that are composed of sp^2^ hybridized carbon atoms arranged in soccer like configuration [1–7]. Fullerenes are allotropes of carbon that occur as spheres of carbon, unlike graphite, that occurs as sheet of carbon. Their name was given because of the resemblance of their structure to the geodesic dome built by Sir R. Buckminster Fuller. The most common fullerene is C_60_, which is called buckminsterfullerene. This buckyball is a molecule with sixty carbon atoms, with each atom present at the vertices of the regular truncated icosahedron (a polygon with 60 vertices and 32 faces). It has 20 hexagonal faces (6 membered rings) and 12 pentagonal faces (5 membered rings) in icosahedral symmetry, forming a closed shell [1–7]. Every ring in this football-like structure is aromatic. In its structure, each carbon atom is trigonally bonded to the other carbon atoms, and the nearest distance, calculated via nuclear magnetic resonance (NMR), between the neighboring carbon atoms is 1.44 angstrom (which is identical to graphite) [1–7]. The four valence electrons of carbon are involved in the formation of two electron-poor single bonds and one electron-rich double bond. As the carbon in this molecule has its valence shells satisfied, the as-formed soccer like structure is a van der Waals solid with a non-conducting nature (an insulator or a semiconductor) [1–7]. Surface functionalized fullerenes with functional groups bearing hydroxyl, carbonyl, etc. exhibit enhanced aqueous solubility [1–7]. These can also be functionalized with various bioactive molecules to achieve targeted delivery and therapeutic outcome. Since its discovery in 1985, this soccer ball like nanomaterial has triggered research on carbon-based nanomaterials for various biomedical and non-biomedical applications [1–7]. It is the third allotrope of carbon after graphite and diamond and can be synthesized via various modern techniques such as arc discharge, laser ablation, etc. [1–7]. These carbon nanomaterials have shown growing potential and relevance in biology (as biosensors, in drug delivery and in nanomedicine), energy storage devices, and photovoltaics due to their unique electronic and above-mentioned structural properties (Figure 1) [1–7].

## 2. Neuroprotective Effect of Fullerenes

**Figure 2.**
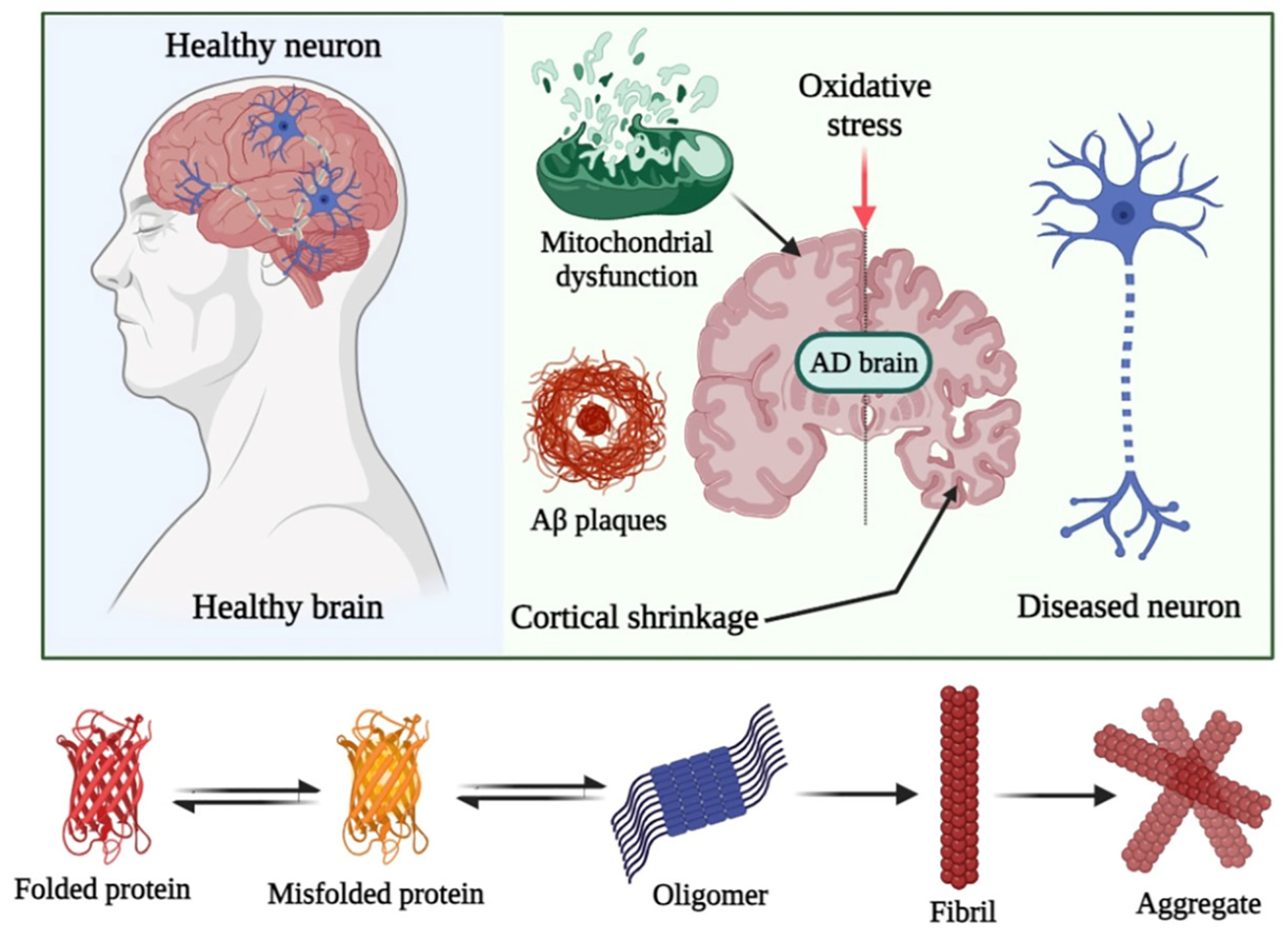
Trajectory of amyloid aggregation induced by oxidative stress and the subsequent neuronal and cognitive decline.

Neurodegeneration refers to a wide range of conditions that result from the progressive loss of structure or function of neurons over time. The most common type of neurodegenerative disorders includes Alzheimer’s disease (AD) and Parkinson’s disease (PD) [8,9]. They affect millions of people worldwide, and are among the leading causes of dementia and death in people aged 65 years and older [8,9]. Unfortunately, early diagnosis and timely efficient treatment remain difficult to achieve in these disorders. The accumulation of abnormal protein aggregates such as alpha synuclein in PD and beta amyloid and tau protein (which forms fibrils upon misfolding and aggregation) in AD, is characteristic in the pathology of these neurodegenerative disorders. Studies have shown a direct link between Aβ fibrillation and oxidative stress [8,9]. It is known that Aβ induces increased production of reactive oxygen species (ROS) and damages mitochondria, which are the powerhouses of cells. These interactions can trigger apoptosis, which is considered as the major type of cell death in AD and PD (Figure 2) [8,9]. Therefore, a drug that can alleviate ROS production and inhibit amyloid fibrillation could be an ideal candidate for treating neurodegenerative diseases.

There are currently over 180 drugs being studied and at various stages of development. However, because of the intricate nature of AD and PD and the diverse characteristics of the affected populations increased efforts must be made to address the multifaceted challenges presented by these conditions and to accommodate the inherent heterogeneity within the affected populations [10,11]. The primary focus of most of these drugs is symptom management and only a minority aim to prevent or arrest neurodegeneration. Therefore, the development of drugs with dual properties is advancing at a very unsatisfying rate. Table 1 highlights some of the current treatments for AD and PD.

Since their discovery, fullerenes and their derivatives have drawn considerable attention in the biomedical field, owing to their excellent antioxidant and neuroprotective potential [21–24]. Because of their ability to interact with and influence assembly of peptides and proteins, fullerenes and their derivatives have the potential to serve as novel therapeutics in treating neurodegenerative disorders [25]. The present review focuses on two of the several molecular outcomes associated with AD and PD and summarizes the role of fullerenes in mitigating the deleterious effects, including cell death arising from amyloid aggregation and oxidative stress.

### 2.1. Potential of Fullerenes and Their Derivatives in Mitigating Amyloid-Associated Toxicity

**Figure 3.**
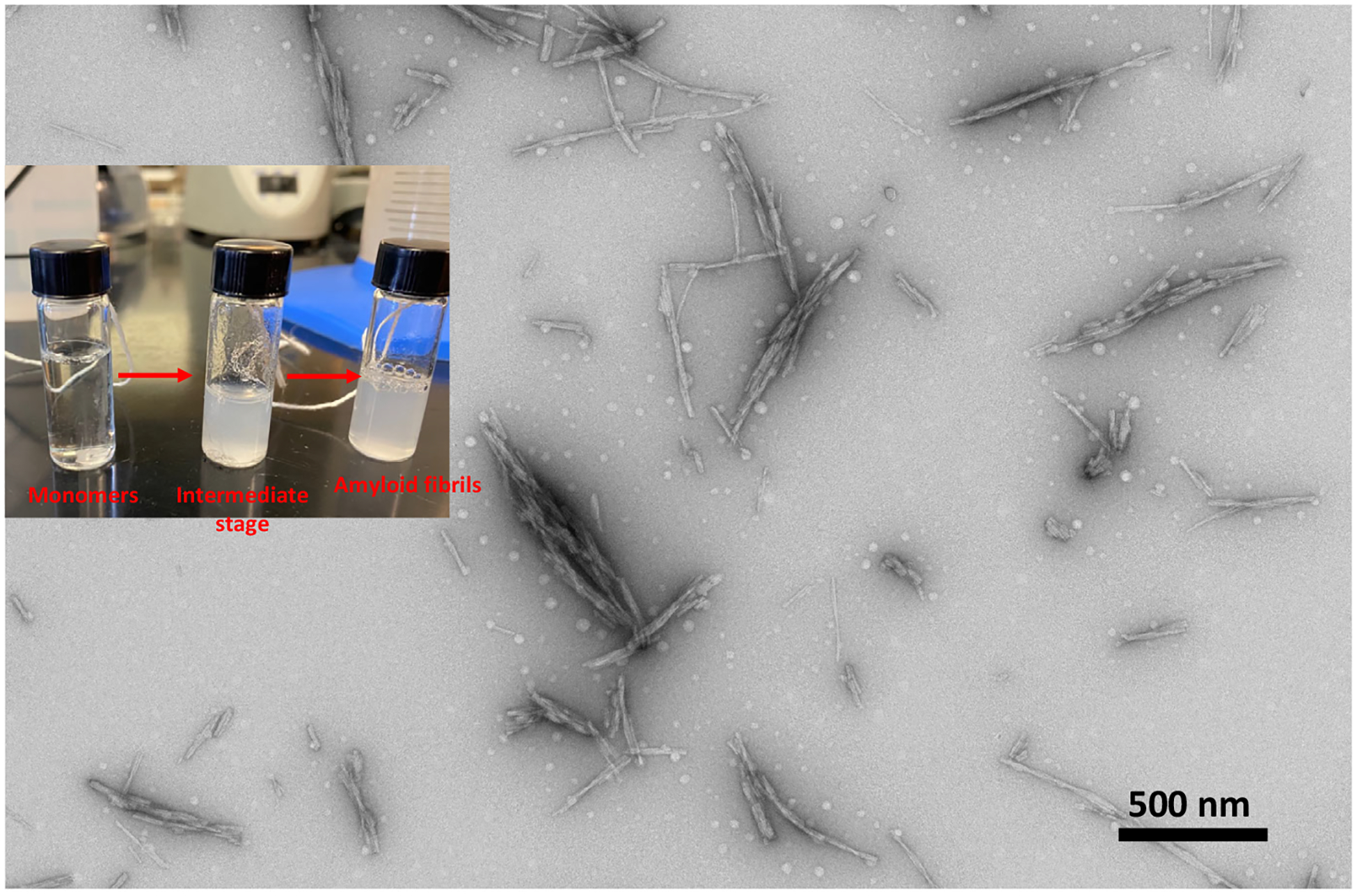
TEM image of lysozyme fibrils produced in the lab of Dr. Narayan. The inset shows an image of various stage of amyloid during fibril formation.

Amyloid fibrils are formed via a complex multistep process involving self-assembly of misfolded amyloid proteins into a soluble oligomer. Oligomers, regarded as primary neurotoxic agents, can then form insoluble beta-sheet rich oligomers, protofibrils, mature fibrils and plaques. Therefore, aggregation of protein from free monomeric amyloid beta to a fibrillar state involves numerous intermediate stages including nucleation, elongation (oligomerization and protofibril formation) and saturation (fibril and plaque formation) [25–30]. Despite differences in the amyloid polypeptide precursor, the resulting amyloid fibrils share common/generic features including well-defined cross beta-sheet structures with beta sheets running parallel to the fibril axis, insolubility due to alpha-helical to beta fold transition and specific staining with thioflavin T (ThT) and Congo red dyes. More than fifty human amyloid misfolding diseases have been identified to date [25–30] (Figure 3).

The amyloid fibrils are implicated in several disease conditions, such as AD and PD. Aggregation of these insoluble amyloids can induce toxicity or interfere with normal functioning of cells, resulting in the progression of disease [25–30]. This fibrillization process occurs due to the imbalance between the production and removal of amyloid beta in brain vasculature and parenchyma [8]. Amyloid beta deposition represents the pathological hallmark of disease and is responsible for neuronal loss, vascular damage, neurofibrillary tangle formation, and dementia. The presence of amyloid fibrils in affected tissues indicates a disease condition. [25–30]. In vitro, the fibrillization process can be affected by several factors including solution properties such as ionic strength, temperature, pH, +/− of chaperones and inhibitors. An abundance of research indicates that nanoparticles can interfere in amyloid formation. However, whether nanoparticles accelerate or decelerate the process of fibrillization is still controversial, and it depends on the physico–chemical properties of nanoparticles and stability of the protein [25–30]. For example, if the mutant (a protein that is easier to misfold or aggregate) has high intrinsic stability and a low intrinsic aggregation rate then nanoparticles will accelerate the process of fibrillization, whereas if the intrinsic stability of the mutant is low and its intrinsic aggregation rate is high, opposite trends are observed, where nanoparticles tend to retard amyloid fibril formation. Therefore, study of the biological applications of fullerenes has attracted increasing attention, which is especially promising in the field of neuroprotection [25–30].

In this section, we highlight and summarize research demonstrating inhibitory and therapeutic ability of fullerenes and their derivatives in amyloid beta aggregation trajectory. This section will highlight how hydrophobic, amphiphilic and hydrophilic fullerenes have different mechanisms of action, either disintegrating pre-formed amyloid fibrils or interfering/inhibiting formation of amyloid fibrils. As summarized here, research demonstrates that fullerenes and their derivatives could inhibit or disintegrate amyloid aggregation via interfering with structure stabilizing interactions such as hydrophobic interaction and salt bridges within the amyloid fibrils.

Sun et al. conducted an atomistic simulation to study the effect of 1,2-(dimethoxymethano) fullerenes (DMF) on amyloid beta (Aβ) aggregation [31]. Their results showed that the interaction between DMF and Aβ resulted in the distortion of β hairpin structure and inter-peptide β sheets structures within the amyloid fibril. In addition to the interaction between the hydrophobic core of the Aβ, leucine–valine–phenylalanine–phenylalanine–alanine (LVFFA), the DMF also interacted with the aromatic residues, namely, phenylalanine 4, tyrosine 10 and C-terminal hydrophobic stretch isoleucine 31–valine 40. Hence, the results obtained from the simulation provide information about the possible interactions between the DMF and Aβ fibril that might be responsible for the inhibition of amyloid aggregation [31].

In another study, water-soluble fullerenol C_60_(OH)_16_ was synthesized to prevent Aβ fibrillation. The inhibition of amyloid formation was followed using the thioflavin T (ThT) assay and atomic force microscopy (AFM) images [32]. Simulation studies were performed to study the possible interactions between the amyloid and the fullerenol. The simulation results show that the electrostatic interactions between the hydroxyl group of fullerenol and the carboxyl group of the amino acids and the hydrophobic interaction between fullerenol and C-terminal of the peptide were responsible for preventing the self-assembly of the peptide and for the structural disruption of the Aβ fibril. To assess the toxicity profile of the fullerenol, a cell viability assay was performed on neuroblastoma cells with no observed significant toxicity. Thus, the results show the potential of fullerenol as a therapeutic drug for AD [32].

In a different study, the potential of hydrophobic fullerene to inhibit formation of β-sheet rich oligomers was evaluated using an atomistic simulation study [33]. The results showed that fullerene was able to prevent β-sheet rich fibril formation, composed of glycine–asparagine–asparagine–glutamine–glutamine–asparagine–tyrosine (GNNQQNY), by strongly interacting with the nonpolar amino acids N3, Q4 and Q5, thus increasing the exposure of the peptide backbone to water and hence preventing the inter-peptide N3–Q4, Q4–Q4 and Q4–Q5 interactions that are crucial for the β-sheet formation and oligomerization. Hence, from the obtained results, it can be concluded that fullerenes can act as potential therapeutic candidates against amyloidosis [33].

Melchor et al. studied the ability of diethyl fullerenemalonates to inhibit Aβ_42_ aggregation [34]. The dose-dependent inhibitory activity of as-synthesized fullerenes was studied using the ThT assay and transmission electron microscopy (TEM). The cytotoxicity of the drug was tested on neuroblastoma cells, which displayed no significant toxicity, thus rendering the fullerenes biocompatible. Hence, from the obtained results, it can be concluded that fullerenemalonates can serve as a future therapy to treat AD and other types of dementia [34].

**Figure 4.**
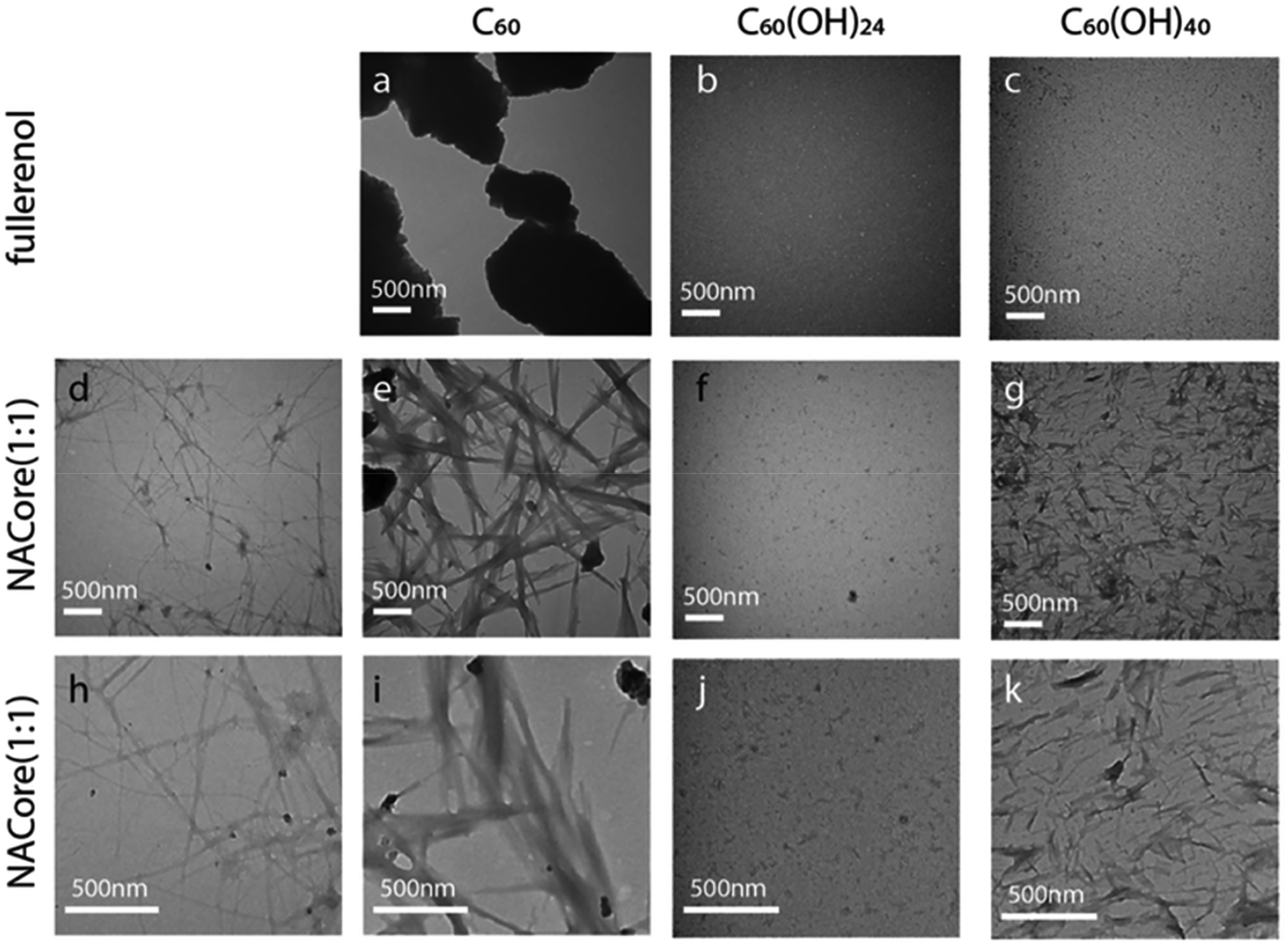
NACore aggregation morphology probed by TEM imaging. TEM images of (**a**) hydrophobic C_60_, (**b**) amphiphilic C_60_(OH)_24_, and (**c**) hydrophilic C_60_(OH)_40_. NAcore aggregates in the absence (**d**,**h**) and presence of (**e**,**i**) C_60_, (**f**,**g**) C_60_(OH)_24_, and (**j**,**k**) C_60_(OH)_40_. Reproduced with permission from Ref. [35].

In another study, fullerenol of variants C_60_ (hydrophobic), C_60_(OH)_24_ (amphiphilic) and C_60_(OH)_40_ (hydrophilic) was tested for its inhibitory activity against amyloid aggregation [35]. Due to their aggregative properties, and thus reduced surface area, the C_60_ hydrophobic fullerenes were not able to prevent self-assembly and hence aggregation of the amyloidogenic core region of the non-amyloid-β component in alpha-synuclein (NACore). Despite the formation of aggregates in the C_60_(OH)_24_ amphiphilic fullerenol, hydroxyls on the surface still allowed for interaction with the peptide backbone of the amyloid, thus interrupting the formation of β-sheet rich aggregates. On the other hand, the C_60_(OH)_40_ hydrophilic fullerenol, although effective at reducing the formation of amyloid aggregation, did not significantly interact with the backbone peptides, indicating that an increase in hydroxyls does not necessarily enhance the interaction with peptides to reduce amyloid aggregation. As a result, both β-sheet rich aggregates and β-barrel intermediates were significantly impacted and hence suppressed, unlike in the case of hydrophobic fullerenes. The observed interaction between the polar regions of the fullerenol and the peptide backbone of the amyloid provide invaluable insight that could be essential in the future development of theranostics. This inhibition or suppression was followed using ThT assay, TEM, FT-Infrared spectroscopy (FTIR), and computational studies. Hence, it can be suggested that fullerenol C_60_ (OH)_n_ with *n* = 0, 24 and 40 can be used as an anti-amyloid inhibitor to treat PD [35] (Figure 4).

Podolski et al. studied the effect of hydrated fullerenes on Aβ_25–35_ fibrillization, both in vitro and in vivo [36]. Images from TEM showed inhibition of Aβ_25–35_ fibrillization in the presence of hydrated fullerenes. In vivo data showed improved cognitive performance in rats upon a single intracerebroventricular injection of hydrated fullerenes compared to rats injected with an Aβ_25–35_ insult. Hence, hydrated fullerenes can be useful in developing AD therapy [36].

Andujar et al. studied the effect of fullerenes on pentameric construct of Aβ units, a model used for studying Aβ fibrillation [37]. The authors discovered that fullerenes, upon interacting with Aβ pentamer, caused alteration in its secondary structure. These changes in key interactions in the structure of wild type Aβ pentamer include destruction of the helical twist and loss of structure stabilizing forces such as hydrophobic interaction (near the turns) and salt bridges. The results of these molecular dynamic simulations demonstrate the ability of fullerenes to destabilize native fibril structure and point towards their ability to serve as inhibitors of fibril formation in AD [37].

Bobylev et al. investigated the effect of sodium fullerenolate (NaFL) on Aβ_1–42_ fibrillization [38]. The cytotoxicity of NaFL was tested across nine cell lines where negligible toxicity was recorded. In vivo tests in mice using intraperitoneal injection revealed low toxicity and low acute toxicity, with the maximum tolerable dose corresponding to 1000 mg/kg. TEM data coupled with fluorescence analysis showed that the NaFL: protein ratio 1:1 (*w*/*v*) disintegrated mature Aβ_1–42_ fibrils. Also, an inhibitory effect was observed where Aβ_1–42_ did not fibrillize, even after 24 h in the presence of NaFL. These effects make NaFL therapeutically interesting for neurodegenerative disorders [38].

Xie et al. studied the effect of fullerenes on β-sheet formation in Aβ_16–22_ peptides using replica exchange molecular dynamics (REMD) and AFM experiments [39]. The obtained data showed that the C_180_ molecule (albeit with same number of carbon atoms as three C_60_ and can be represented as 3C_60_) showed great inhibitory effect on β-sheet formation in Aβ_16–22_ peptides. The driving force behind this effect was hydrophobic and pi-stacking interactions between C_180_ and Aβ_16–22_ peptides. These interactions weakened peptide–,peptide interactions which are crucial for β-sheet formation, thus retarding Aβ_16–22_ fibrillization. Overall, the finding provide insight into fullerenes’ ability to be developed as drug candidates against AD [39].

Zhou et al. investigated the inhibitory effect of water-soluble fullerene derivative on aggregation of fibrillar Aβ_1–42_ hexamer (a protofibril model) via all-atom explicit solvent molecular dynamics (MD) simulations [40]. Fullerene 1,2-(dimethoxymethano) (DMF) binds to Aβ protofibrils at multiple sites within a 90 ns time scale. The three identified binding sites were the hydrophobic core, turning site and C-terminal β-sheet site. The binding interactions between DMF-Aβ revealed hydrophobic and pi-stacking as the predominant binding mechanisms. Binding to the turn region of Aβ can disrupt salt bridge formation, which is important for Aβ fibrillation. These findings provide molecular insights into the interactions between Aβ and DMF and showcase the potential of fullerene derivatives as therapeutic drugs against AD [40].

### 2.2. Potential of Fullerenes and their Derivatives in Mitigating Oxidative Stress

Many neurodegenerative disorders arise due to the imbalance in the production and removal of ROS and reactive nitrogen species (RNS) or due to alteration in the functionality of the antioxidant defense system of the cells. These alterations can arise either from mutations in radical scavenging enzymes such as superoxide dismutase (which catalyzes the conversion of superoxide radical into hydrogen peroxide), glutathione peroxidase and catalase (which catalyzes the conversion of hydrogen peroxide to water molecules) or due to exposure to environmental toxicants such as pesticides [41–43]. The brain is vulnerable to oxidative damage and consumes 20% of all oxygen and 25% of all glucose intake in the body. The main source of ROS is the electron transport chain (ETC) in the mitochondrial membrane where ATP is generated. These superoxide and nitric oxide radicals can also originate from overexcited glutamic acid receptors, astrocytes, and microglia. The ROS/RNS can be produced via Fenton chemistry, as the brain has a high content of redox active metals. These free radicals can attack biological molecules such as DNA, RNA, lipids, carbohydrates and protein, causing their oxidation. These modifications in their external structure can then produce more potent oxidants. Oxidation of DNA or RNA introduces nucleic acid strand break, which can affect crucial gene replication, transcription, and translation. Oxidation or carbonylation of proteins can lead to protein misfolding and biologically unfunctional protein, which can initiate diseases such as amyloidosis [41–43]. The brain consumes a high content of polyunsaturated fatty acids (PUFA) and these are sensitive to oxidation. Therefore, oxidation of lipids (peroxidation of PUFA) can impact structural integrity of the cell membrane which can result in cell apoptosis. Hence, fullerenes, due to their sp^2^ hybridized architecture and therefore their ability to act as free radical sponges, have demonstrated promising behavior in this field [41–43].

**Figure 5.**
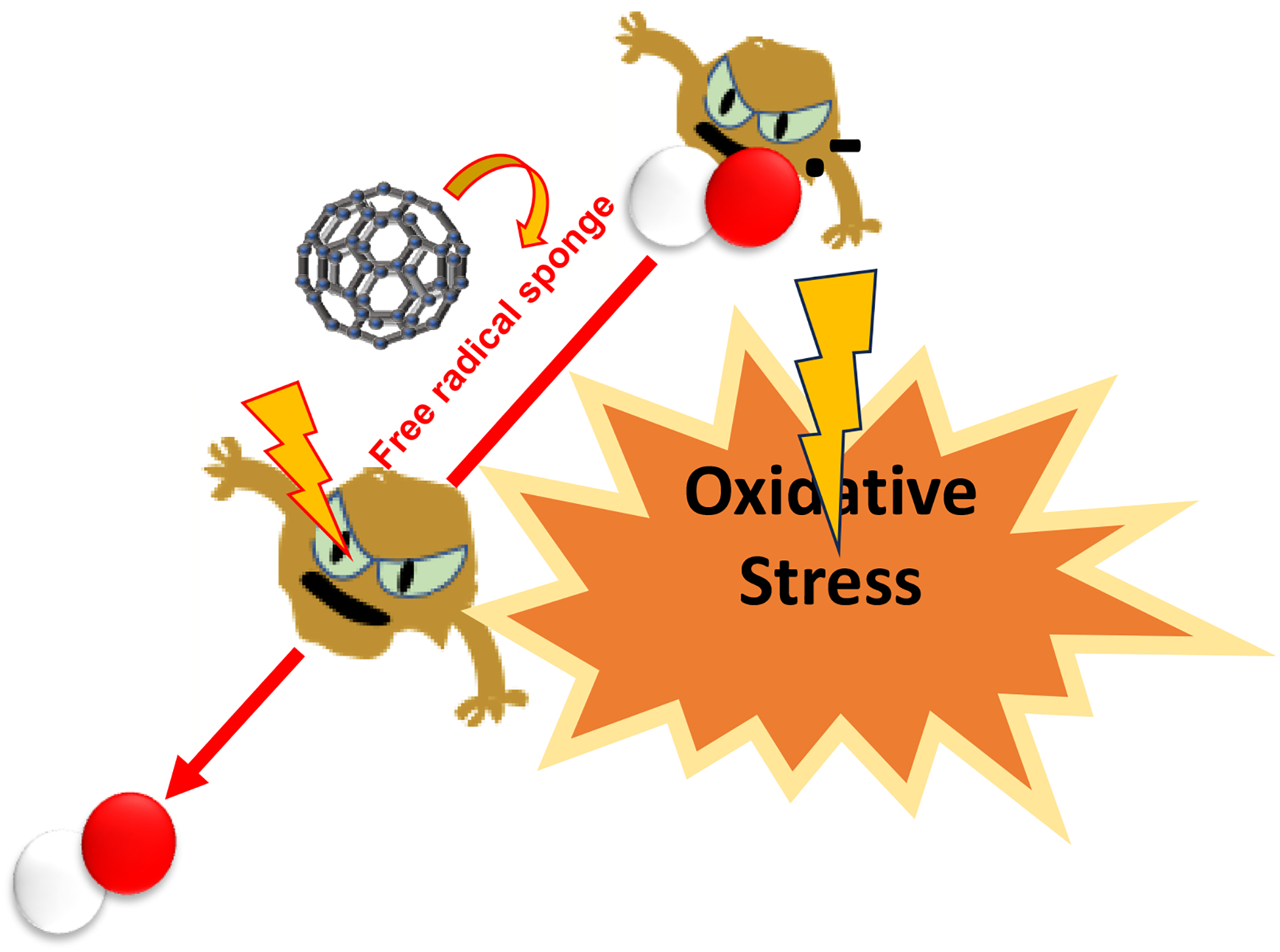
Schematic representation of the antioxidative effect of fullerenes.

This section highlights and summarizes research pertaining to antioxidant potential of fullerenes and their derivatives. It demonstrates how either PEGylation or introduction of a carboxylic group to fullerenes improves their free radical scavenging abilities (Figure 5).

In another study, pentoxifylline-C_60_ (PTX-C_60_) nanoparticles were synthesized to overcome the Aβ_25–35_ associated cytotoxic effects in Neuro-2A cells. The formulation significantly reduced the Aβ_25–35_ induced neuronal death by rescuing the cells from oxidative stress, decreasing the ROS levels and maintaining the mitochondrial membrane potential (MMP) [44].

In another study, PEGylated-C_60_ was prepared as a radical sponge to mitigate the neuronal apoptosis induced by Aβ_25–35_. Endoplasmic reticulum (ER) stress response genes and antioxidant related genes were analyzed to study the response of the C_60_ against Aβ_25–35_ induced cytotoxicity. The results showed the protective activity of C_60_ against Aβ_25–35_ treatment in Neuro-2A cells [45].

In a different study, carboxylic acid functionalized C_60_ were synthesized as free radical scavengers of cultured cortical neurons against N-methyl–D-aspartate and alpha-aminoso-3–hydroxy-5–methyl-4–isoxazoleproponic acid, thus pointing towards the potential of these water-soluble C_60_ derivatives as active therapeutic agents against several acute or chronic neurodegenerative diseases [8]. The protective efficiency of C_60_ against Aβ_25–35_ induced neurotoxicity upon intrahippocampal microinjection in mice was analyzed in a different study. The results were impressive, as the introduction of buckyball before the causative agent’s introduction was able to prevent any disturbances to protein synthesis, thus pointing towards the possibility of developing an anti-amyloid drug with free radical scavenging capability and anti-aggregative capabilities [46].

In a final study, Du et al. synthesized a near-infrared-switchable nanoplatform for the treatment of AD [47]. In this study, upconversion nanoparticle (UCNP@C_60_) was prepared and functionalized with the amyloid fragment KLVFF, resulting in the generation of dual property UCNP@C_60_–KLVFF. Under the near-infrared light (NIR), this nanoparticle produced ROS that caused photooxygenation of the amyloid peptide. This oxidation of the amyloid, in turn, prevented the protein aggregation through the covalent addition of oxygen onto the hydrophobic cluster of Aβ. In the absence of the NIR, the UCNP@C_60_ acted as a ROS scavenger by quenching the free radicals and relieving the oxidative stress and the associated cytotoxicity, thus enhancing the longevity of *C. elegans*. Therefore, it appears that this nanoplatform can be used as a theranostic and can provide synergy therapy for AD patients [47].

## 3. Outlook and Conclusions

Fullerenes, owing to their unique properties such as redox activity, as they have a low LUMO level and high HOMO level, are now being widely used in several areas of science, particularly in biomedicine [48,49]. These unique properties have opened up various biomedical applications of fullerenes, such as biosensors, radiopharmaceuticals or drugs, targeted drug delivery systems and the carrying of contrast agents. Different therapeutic strategies have been used for targeting amyloid beta production and clearance. However, microhemorrhages and disruption in important metabolic processes remain an issue, which has resulted in the failure of these strategies [48,49]. Therefore, targeting/inhibiting amyloid peptide self-assembly or disintegration of the pre-existing fibrils is another approach that could have potential for neurodegenerative disorder therapy [48,49].

The neuroprotective ability of fullerenes can be attributed to their small size (less than 1 nm in diameter), and thus increased accessibility within the membrane, as well as antioxidant properties due to their high reactivity towards free radicals, their unique structure, promoting high electrophilicity and strong hydrophobicity, and their ability to cross the blood brain–barrier [50–52].

Depending on their composition, charge, shape, and size, fullerenes and their derivatives have been reported to affect the fibrillation process differently. In addition to dissolving already formed fibrils and preventing the formation of fibril by arresting/interacting with the intermediate species in the aggregation pathway, fullerenes can also be fine-tuned to detect the formation of amyloid aggregates and provide mechanistic understanding of the aggregation pathway [48,49]. One of the major hinderances that limits the biomedical application of the fullerenes is their poor water-solubility and toxicity to cells at higher doses [53–55]. Fullerenes could be important molecules in the treatment of neurological disorders, but their molecular design needs further investigation. For example, surface functionalization that attaches hydroxyl groups to the carbon cage could be performed for designing water-soluble derivatives of C_60_ [56]. Critical evaluations must take place, during and after the synthesis of fullerenes, as morphology is the primary factor that dictates their usability for specific biomedical applications, and this is therefore a major influence on the toxicological profile of the fullerene [57]. Furthermore, investigation into the metabolic reactions could also be useful, to understand their reactivity with biological systems [58–61]. An in-depth examination of the harmful effects of fullerenes on cell proliferation, their ability to induce cancer, etc., is crucial for increasing their usefulness in the treatment of neurodegenerative disorders [58–61].

## Figures and Tables

**Table 1.** Current treatments for Alzheimer’s disease and Parkinson’s disease.

Neurodegenerative Disorder	Drug Class	Drug Indications	Mechanism of Action	Drug Name	Reference
Alzheimer’s Disease	Cholinesterase inhibitor	Increases cognitive function by increasing levels of acetylcholine	Prevents the hydrolysis of acetylcholine into acetate and choline	Donepezil Rivastigmine Galantamine	[12]
	N-Methyl-D-Aspartate (NMDA) receptor antagonist	Regulates glutamate activity and prevent exitoxicity	Binds to NMDA receptors and reduces the influx of Ca+ to regulate glutamate activity	Memantine	[13]
Parkinson’s Disease	Dopamine agonists	Increases dopamine availability	Activates dopamine receptors D2 and D3 receptors	Pramipexole Apomorphine Transdermal Ritigotine Ropinirole	[14]
	Levodopa	Manage motor symptoms	Decarboxylases into dopamine	Carbidopa	[15]
	Monoamine oxidase type B (MAO-B) Inhibitors	Increases dopamine availability	Inhibit the deactivation of dopamine	Selegiline Rasagiline Safinamide	[16]
	Cathechol-O-Methyl transferase (COMT) Inhibitors	Manage motor symptoms when used with levodopa	Inhibit COMT activity to reduced the methylation of catecholamines	Tolcapone Entacapone Opicapone	[17]
	Adenosine 2A Antagonists	Manage motor symptoms when used with levodopa	Inhibit A2A receptor antagonists	Istradefylline	[18]
	Anticholinergics	Manage motor symptoms	Inhibit binding of neuro transmitter acetylcholine	Trihexyphenidyl Benztropine Orphenadrine Procyclidine Biperiden	[19]
	Amantadine	Used as a prophylactic while taking levodopa	Inhibits the re-uptake of N-methyl-D-aspartate antagonism	Gocovri Symmetrel	[20]
